# COVID-19 as a “Digital Push?” Research Experiences From Long-Term Care and Recommendations for the Post-pandemic Era

**DOI:** 10.3389/fpubh.2021.660064

**Published:** 2021-05-10

**Authors:** Vera Gallistl, Alexander Seifert, Franz Kolland

**Affiliations:** ^1^Institute of Sociology, University of Vienna, Vienna, Austria; ^2^Centre for Gerontology, Karl-Landsteiner-Privatuniversität, Krems, Austria; ^3^School of Social Work, University of Applied Sciences and Arts Northwestern Switzerland, Olten, Switzerland

**Keywords:** inequality, digital divide, gerontology, long-term care, ICT

## Background

Health authorities and governments worldwide label older adults as a “risk group” for more serious and possibly fatal illness associated with SARS-CoV-2 (COVID-19) (1). Those older adults living in long-term care (LTC) are considered especially prone to more serious and fatal disease following a COVID-19 infection (2). Consequently, measures requiring LTC residents to shelter in place and maintain physical distancing from others have been enacted in many countries during the pandemic. However, these measures make this group particularly prone to isolation, especially because the measures have prevented family members from visiting nursing homes for several months. Researchers have posed concerns that these particularly strict measures are likely to enhance feelings of loneliness and isolation in LTC residents (3).

The use of digital technologies by LTC residents has been painted as a hopeful alternative to face-to-face contact during the COVID-19 pandemic. Several digital solutions have been suggested to decrease the social isolation of older adults, such as Skype, FaceTime, or Zoom, which allow LTC residents and their families to interact virtually (3). Indeed, the use of information and communication technologies (ICT), such as the Internet, offers many benefits (4) for older adults living in nursing homes who wish to stay in contact with others while following COVID-19 social distancing measures. The Internet has particularly been shown to play an important role in distance-based social contact during the coronavirus pandemic (5).

Although ICT use might help older adults maintain social interaction, LTC residents may also feel socially excluded because they lack the necessary skills and equipment to be included in the digital society (6, 7). Studies have shown that nursing home residents are less likely than community-dwelling older people to take advantage of the opportunities provided by modern ICT (8) for the following reasons: they might (a) opt not to use the Internet, (b) live in an environment where Internet access is not available, (c) not have sufficient support from inside or outside their nursing homes, and (d) have physical or cognitive limitations that limit or prevent ICT use without assistance (9, 10). Furthermore, when nursing homes isolate their residents from outside contact, they may prevent individuals from ICT support from outside the facilities. Thus, the tremendous barriers to ICT use in nursing homes are not likely to be handle everywhere during the pandemic, when LTC systems are under a considerable amount of general pressure with coping the current pandemic.

This opinion article investigates and discusses the evidence for a “digital push” in LTC during the COVID-19 pandemic by referring to data regarding Internet use from 259 nursing home residents. Building on these findings, the article provides research and policy recommendations to enhance and support LTC residents' digital engagement during the COVID-19 pandemic and beyond.

## The Digital Divide in Long-Term Care and Its Changes During the Pandemic

Research has shown that more residents of nursing homes are using the Internet; however, this Internet use is at a lower level than that of younger adults (11). Furthermore, specific LTC groups are more like to use ICTs, while others are not likely to engage in ICT use. For example, Seifert and Cotten (8) showed that only 21% of retirement home residents in Zurich (Switzerland) used the Internet. Compared to non-users, Internet users were more likely to be younger, healthier, and more functionally unimpaired (8). Of the participants in this study, only 12.8% reported owning a smartphone, and even fewer reported owning a tablet (4.5%). Similar results were generated by a study in Germany that involved 1,863 people aged 80 years and older who lived in private households and LTC facilities (11). Only 3% of the participants in LTC facilities reported using Internet-connected ICT devices. ICT device adoption was associated with functional health, chronological age, education, and interest in technology (11). The results of these studies showed that a minority of LTC residents used ITCs before the pandemic and that the more educated, younger, and healthier residents were more likely to use ICTs.

However, current research also showed that institutional context and ICT infrastructures in nursing homes are important aspects of digital engagement in LTC (10, 12). Studies conducted before the COVID-19 pandemic found that ICT availability is often limited in LTC facilities, which have a significant deficiency of ICT infrastructures (13, 14). This deficit also includes a lack of ICT skills among the care staff, as well as the staff's reserved attitude toward technology use within LCT facilities (15). The COVID-19 pandemic further complicates this complex relationship between individuals' ability to use ICT and the institutional infrastructures that govern the access to ICT in LTC. The ongoing pandemic has created new awareness of the existing limitations of these facilities' current ICT infrastructures (16–18), but it has also restricted opportunities to remove these institutional and individual barriers to ICT.

## Research on Internet Use in Long-Term Care During the Pandemic

Between August and September 2020, we included some ICT-related questions in a bigger study about the coping strategies of residents living in nursing homes during the pandemic (19). The representative sample included 259 residents from 16 nursing homes evenly distributed across Austria. The participants, who were all LTC residents for at least 3 months before the study, filled out a standardized questionnaire with closed questions. The age range of the sample was 47–101 (mean age: 83 years). Of the respondents, 74% were female, and 26% were male. About one-third (32%) had lived in LTC less than a year, while another third (32%) had lived in LTC for more than 4 years. In total, 56% of the respondents reported multiple limitations in daily living activities (e.g., personal hygiene, getting dressed, eating, taking a walk).

Concerning the digital engagement of the LTC residents during the COVID-19 pandemic, only a small percentage (9.2%) of the respondents reported using the Internet (4.1% “often” and 5.1% “sometimes”) to stay in contact with their relatives. ICTs seemed to be particularly unpopular; the majority of respondents (99.3%) used the telephone to stay in contact with their families. While a need for more personal contact was apparent—half of the respondents (49%) stated that they felt lonely often (17%) or occasionally (32%)—digital solutions to combat this loneliness did not seem to be an option for residents in LTC.

The data also revealed that although levels of loneliness had remained high during the COVID-19 pandemic, ICT use had hardly increased among the respondents. When asked about the increase or decrease of Internet use during the pandemic-related lockdown in Austria (i.e., when contact restrictions were in place), 33.3% of the 42 residents (33.1% of the whole sample) who reported using the Internet stated that they used it more often during the lockdown, 31.3% used it the same amount, and 35.7% used it less often. In comparison, 37.1% of the telephone users (*n* = 250) reported using the telephone more often to stay in contact with relatives, while 55.5% of the Internet users (*n* = 44) reported using the Internet more often for this purpose.

Several assumptions can be made about this relatively small increase in digital engagement during the COVID-19 pandemic. First, as stated before, the ICT infrastructure and Internet use among LTC residents were both limited before the pandemic. Second, the ICT skill set of LTC residents may be lower than younger people; therefore, learning new ICT skills is more time-consuming for residents. Third, during the pandemic, LTC facilities do not have the personal resources necessary for organizing new ICT hardware for the residents or helping the residents learn ICT use. Finally, ICT support (e.g., from family and friends) from outside the LTC facilities was limited during the pandemic.

## Recommendations for the Post-Pandemic Era

As seen above, an easy “parachuting in” of technology during the pandemic is not likely in LTC, mainly because the Internet is not the main medium for social interactions for older adults aged 80 years and older (11). A study in the US found that 27% of people aged 65 years or older still did not use the Internet, compared to <10% of adults under that age (20). In contrast, a representative survey conducted across European Union countries showed that 49% of people aged 50 years or older used the Internet (21); the same study showed that people older than 80 years spend less time online than people in the next highest age group (65–79 years) and that men and older adults with a higher educational or economic status are more likely to use the Internet. Furthermore, individuals' health, prior experience with technology, social salience (Internet use among the members of their social network) and contextual factors, such as country-specific wealth and communication technology infrastructure, are predictors of Internet usage by older adults. Similar differences in the use of the Internet are seen with other emerging technologies (22, 23). Furthermore, institutional mechanisms in LTC are not sufficient for supporting older adults' appropriation of digital technologies (8, 9, 12).

Based on the presented data from Austria, a simple “parachuting in” of digital technologies in LTC will not be enough to ensure sustainable engagement with ICT that actually prevents loneliness and social exclusion during the COVID-19 pandemic. Internet use in LTC is a complex and relational process that is highly dependent not only on the interest and motivation of residents but also, and even more importantly, on institutional mechanisms, support structures, and opportunities.

Therefore, the hope in a “digital push” spurred by the COVID-19 pandemic may be just that—optimism in the face of overwhelming social isolation for older LTC residents. In that sense, current discourses surrounding digital solutions that aim to support the social inclusion of older LTC residents must be reconsidered. This consideration should determine whether the current discourses are actually designed to provide help to LTC residents or are part of a general techno-optimism that often accompanies discourses concerning digitalization and later life (24). Techno-optimism characterizes demographic change as a problem and (digital and assistive) technologies as the adequate solution to solve this problem.

Given the rapid expansion of ICT in society, discussion of further recommendations is worthwhile. Based on the discussion and findings outlined above, we recommend the following:

- Understanding digital engagement in LTC as a process, rather than an intervention, that requires continuous engagement and support, as well as adequate infrastructures and skills, for both residents and care staff.
- Developing and implementing a different perspective on digital technologies in LTC that understands technologies not merely as an artifact or an instrument but also as a learning process that needs to be professionally supported.
- Supporting LTC units in building an adequate infrastructure to enable the digital engagement of their residents by producing public policy and applying research projects.
- Giving LTC residents the ICT skills and training that they need by providing free-of-charge learning opportunities and ICT support (e.g., from ICT-trained recreational therapists) within the LTC facilities.

## Conclusion

When the proper support is provided ICT offers unique and innovative opportunities for older adults living in nursing homes. Older adults, their relatives, and their professional caregivers can take advantage of these digital tools to improve their daily lives. However, the use of technology can be challenging, especially when older adults lack access to new tools or digital skills and are pushed into digital solutions, especially in times of social distancing. Developers, practitioners, and researchers in the field must be aware that the appropriation of digital technologies in LTC is a complex process that requires a variety of actors to be successful and sustainable. Digital engagement in LTC calls for appropriate infrastructures that support older adults' engagement in ICT; these infrastructures include adequate training and learning opportunities for residents and care staff, stable Internet connection, and access to adequate devices. Therefore, future studies should provide practical guidelines that consider the older adult user contextually and his/her individual characteristics (25).

## Author Contributions

All authors listed have made a substantial, direct and intellectual contribution to the work, and approved it for publication.

## Conflict of Interest

The authors declare that the research was conducted in the absence of any commercial or financial relationships that could be construed as a potential conflict of interest.
